# Intra-specific variation of sperm length in the malaria vector *Anopheles gambiae*: males with shorter sperm have higher reproductive success

**DOI:** 10.1186/1475-2875-7-214

**Published:** 2008-10-21

**Authors:** Maarten J Voordouw, Jacob C Koella, Hilary Hurd

**Affiliations:** 1 Department of Biology, University of Victoria, PO Box 3020, Station CSC, Victoria, British Columbia, V8W 3N5, Canada; 2 Division of Biology, Imperial College of London, Silwood Park Campus, Buckhurst Road, Ascot, Berkshire, SL5 7PY, UK; 3 Centre for Applied Entomology and Parasitology, School of Life Sciences, Keele University, Staffordshire, ST5 5BG, UK

## Abstract

**Background:**

Intra-specific variation in sperm length influences male reproductive success in several species of insects. In males of the malaria vector *Anopheles gambiae*, sperm length is highly variable but the significance of this variation is unknown. Understanding what determines the reproductive success of male mosquitoes is critical for controlling malaria, and in particular for replacing natural populations with transgenic, malaria-resistant mosquitoes.

**Methods:**

A laboratory population of *A. gambiae *males was tested for intra-specific variation in sperm length. A full-sib quantitative genetic design was used to test for a genetic component of sperm length in *A. gambiae *males and estimate its heritability. This study also tested for a relationship between sperm length and male reproductive success in *A. gambiae*. Male reproductive success was measured as the proportions of inseminated and ovipositing females.

**Results:**

There was intra-specific variation of sperm length in *A. gambiae*. There was no significant genetic variation in sperm length and its heritability was low (h^2 ^= 0.18) compared to other insects. Sperm length was correlated with male body size (measured as wing length). Males with short sperm had significantly higher reproductive success than males with long sperm and this was independent of body size.

**Conclusion:**

This is the first study to demonstrate intra-specific variation in sperm length in *A. gambiae *and that males with short sperm have higher reproductive success. That sperm length influences female oviposition is important for any strategy considering the release of transgenic males.

## Background

Malaria kills up to three million people each year and is one of the most pressing health concerns in the developing world. The *Plasmodium *parasites that cause malaria are transmitted between human hosts by female mosquitoes of the genus *Anopheles*. One strategy to combat malaria is the release of genetically modified mosquitoes that are incapable of transmitting human *Plasmodium *parasites. Proof of concept was recently demonstrated in the laboratory with a transgenic, rodent malaria-resistant strain of *Anopheles stephensi *mosquitoes [1,2]. Field releases of transgenic mosquitoes will likely involve males, as releasing females would increase biting rates and possibly the prevalence of other mosquito-borne diseases [3]. As the success of the strategy depends upon the reproductive success of the transgenic male mosquitoes, it is important to study the reproductive biology of male anopheline mosquitoes [4].

In *Anopheles gambiae *and other anopheline mosquitoes, males initiate swarms of twenty to thousands of individuals shortly before sunset to attract females [5-8]. During copulation males transfer sperm as well as male accessory gland secretions (MAGS), which inhibit sexual receptivity and induce oviposition behaviour in females [9]. After mating, females leave the swarm [8]. Therefore, polyandry (female multiple mating) is rare in the field [< 3%; reviewed in [10]] and thus post-copulatory sperm competition is probably not important in anopheline mosquitoes [11]. Males return to the swarm after mating [8] and polygamy (male multiple mating) probably occurs in the field, although it has only been demonstrated in the laboratory [12]. Females store the sperm in their single spermatheca and can lay up to 12 batches of eggs in the field [13] with an average of ~100 eggs per batch. In the laboratory, *A. gambiae *females produce a batch of eggs every 3 days when given regular access to blood meals [14].

Voordouw and Koella [15] used a classic quantitative genetic approach to demonstrate genetic variation in male reproductive success in a laboratory population of *A. gambiae*. Full-sib families of males differed significantly in their ability to induce females to oviposit and in the likelihood that their partner's eggs hatched [15]. In another population of *A. gambiae*, Voordouw *et al *[16] showed that oviposition success among groups of males was correlated with the motility of the sperm stored in the females' spermathecae 14 days after mating. Although the significance of this sperm motility is not known, this result suggests that phenotypic variation in sperm influences variation in female oviposition behavior in *A. gambiae*.

Klowden and Chambers [17] demonstrated that males of *A. gambiae *have more variation in sperm length than the males of other anopheline species. Because they combined the sperm of three to five males for each species, it was not clear whether this variation was due to differences among males or to differences among sperm within males. In *A. gambiae*, they also found that the mean sperm length in the genital tract and spermatheca of the female was longer than that in the testes of the male [17]. However, their conclusion that larger sperm are more fertile is questionable, because different males were used to estimate the distribution of sperm lengths in the testes and female reproductive organs.

Intra-specific variation in sperm length has been demonstrated in numerous animals, including several species of insects [18]. Quantitative genetic and selection experiments have shown that sperm length is a heritable trait in the dung fly, *Scathophaga stercoraria *[19], and in the cricket, *Gryllus bimaculatus *[20]. The adaptive significance of sperm length varies among taxa [21]. In two species with aflagellate, amoebic sperm – the bulb mite, *Rhizoglyphus robini *[22], and the nematode, *Caenorhabditis elegans *[23] – males with larger sperm have higher fertilization success. In two insects with flagellate sperm – *G. bimaculatus *[24] and the dung beetle, *Onthophagus taurus *[25] – males with shorter sperm have higher reproductive success.

The first objective of this study was to determine whether there was intra-specific variation in sperm length in *A. gambiae *and, for comparison, in *A. stephensi*. The second objective was to test whether the intra-specific variation in sperm length in *A. gambiae *had a heritable component and whether it was influenced by body size (estimated as wing length). The third objective was to test whether sperm length influenced male reproductive success.

## Materials and methods

### General methods

The outbred *A. gambiae *Keele strain [see [26]] and the *A. stephensi *DUB strain were used. Mosquitoes were kept in insectaries maintained at a temperature of 27°C, relative humidity of ~70% and a 12 h:12 h light:dark photoperiod. Adult mosquitoes were kept in 30 cm cubic cages. Adults were fed *ad libitum *on a solution containing 10% glucose, 0.28% streptomycin/penicillin (Sigma-Aldrich, Poole, UK) and distilled water. Larvae were reared individually in 24-well tissue culture plates. Larvae were fed 0.03, 0.04, 0.08, 0.16, 0.32 mg of ground Tetramin™ per individual on days 1, 2, 3, 4, 5 and 0.60 mg every day thereafter. This feeding protocol was used in other studies [15,16] and results in optimal development (~10 days from larva to adult).

### Experiment 1. Intra-specific variation of sperm length in *A. gambiae *and *A. stephensi*

Twenty-five *A. gambiae *and 17 *A. stephensi *males of unknown age and mating history were haphazardly sampled from stock cages in the insectary. Males were sacrificed in 70% ethanol and dissected for their testes. Each testis (labelled A and B) was mounted separately for each male. Each testis was placed in 10 μl of PBS, torn open with a fine needle and the sperm were dispersed before being covered with an 18 mm cover slip. Slides were immediately mounted on a Leica DM IRB inverted fluorescence microscope and sperm were imaged at 200× using phase contrast illumination. For each testis, five fields of view were haphazardly selected and photographed using a Leica DC 300F digital camera. For each field of view, 10 sperm were haphazardly selected and measured using Leica FW4000 imaging software. Although dispersing the sperm on the slide (as above) resulted in hundreds of loose sperm, many sperm were clumped, which made them impossible to measure.

### Experiment 2. Heritability of sperm length in *A. gambiae*

*A. gambiae *females (~one week old) were blood fed on the arms of MJV for 20 minutes and 60 females were haphazardly selected and transferred to individual oviposition cups. For 32 females that laid enough eggs (> 30), the eggs were hatched in small plastic containers (10 × 7 × 5 cm^3^) containing 200 ml of distilled water. Each batch of eggs from the same female is hereafter referred to as a full-sib family. For each full-sib family, between 30 and 48 hatchlings were haphazardly selected, split into two groups (A and B), and each group was reared in a separate tissue culture plate (following the general methods). The order of the 64 tissue culture plates was randomized on trays kept on the same shelf in the insectary. For each tissue culture plate, the pupae were sexed by examining their paddles for the presence of male tarsal hooks and the males were transferred to an emergence cup. Two and six days after the males emerged, one male was haphazardly selected from each of the 64 emergence cups and its sperm was measured as in experiment 1 except that only one testis was mounted. Hence sperm was measured for 32 full-sib families, with two independent environments (the tissue culture plates) per family, with two males per environment (aged 2 and 6 days), with one testis per male, with five fields of view per testis, and with ten sperm per field of view, for a total of 6400 sperm. For all 128 males in experiment 2, the length of one wing was measured as the distance between the allula and the distal fringe using a compound microscope (50× magnification) and an ocular micrometer.

### Experiment 3. Sperm length and male reproductive success in *A. gambiae*

For the 60 females in experiment 2, each female was blood fed a second time for five minutes on the arms of MJV nine days after their first blood meal. Enough eggs (> 30) were obtained for 28 full-sib families and the offspring were reared as in experiment 2 with one exception. In experiment 2, the larvae were transferred to tissue culture plates as soon as they had hatched, whereas in experiment 3, the larvae spent two days in the communal family containers (i.e. at high density) before being transferred. Experiment 3 pupae were sexed and kept in separate emergence cups to ensure that males and females were virgin. From the 28 available full-sib families, 16 full-sib families (hereafter referred to as sire families) were randomly selected. For each sire family, 10 males were haphazardly selected and placed in a mesh-mating cage (20 cubic cm). On day-4 post-emergence, 10 females were added from a different full-sib family (hereafter referred to as the dam family). The males and females were allowed to mate for two days. On day-6 post-emergence, mosquitoes were cooled (4°C) and sexed, and females were separated into oviposition cups. Of the 160 females, 71 survived the cold room (2 to 8 per sire family). This low survivorship was due to anaesthetized females drowning in the water of their oviposition cups, as previous experiments had 100% survivorship when cold-anaesthetized females were transferred to dry containers (M. J. Voordouw, unpublished data). On day-7 post-emergence, these 71 females were blood fed for five minutes on the arms of MJV. Females were allowed to oviposit on day-7 to day-14 post-emergence. On day-15 post-emergence, all females were sacrificed and their sperm motilities were scored as in Voordouw *et al *[16]. A sperm bundle was defined as motile if at least one motile sperm was observed; it was defined as non-motile if no motile sperm were observed. The males were allowed to regenerate their sperm supply for four days after separating the sexes. On day-10 post-emergence, four males were haphazardly selected from each sire family and their sperm lengths and wing lengths were measured as in experiment 2.

### The mean sperm length of *A. gambiae *in the Klowden and Chambers [17] study

In their study of sperm length variation in five *Anopheles *species (including *A. gambiae *but not *A. stephensi*), Klowden and Chambers [17] presented frequency distributions of sperm lengths divided into 50 μm categories but did not give the means. To compare the mean sperm length of *A. gambiae *between this study and their study, their Figure 1[17] was digitized, the number of sperm in each 50 μm category was estimated, and the mean sperm length was calculated to be ~280 μm.

**Figure 1:**
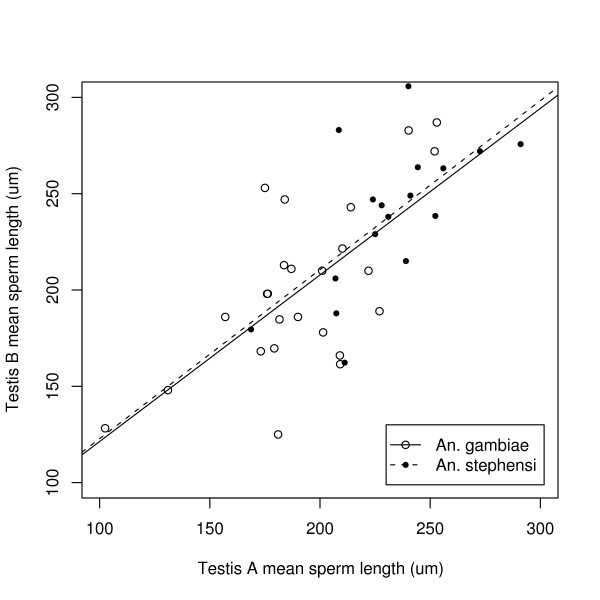
**Intra-specific variation in sperm length in *A. gambiae *and *A. stephensi *males**. Experiment 1: there is significant intra-specific variation in sperm length among males in both *A. gambiae *and *A. stephensi*. The mean sperm length in testis A is strongly correlated with that in testis B in males of *A. gambiae *(n = 25 males) and *A. stephensi *(n = 17 males).

### Statistical methods

#### General statistical methods

The sperm and wing length data in all experiments were normally distributed. The ANOVA function in R, aov, with a nested error term was used to model these data. For sperm length, the factors were nested as follows: sperm within the field of view, fields of view within the testis, testes within the male, males within the tissue culture plate, and tissue culture plates within the family. For wing length, the factors were nested as follows: one wing length measurement for each male, males within the tissue culture plate, and tissue culture plates within the family. F-tests were used to determine the statistical significance of factors in the ANOVA. The linear mixed effects model function in R, lmer, was used to estimate the variance components of sperm and wing length.

#### Experiment 1. Intra-specific variation in sperm length in *A. gambiae *and *A. stephensi*

For each species, a Pearson's correlation test was used to determine whether the mean sperm length in testis A was significantly correlated with that in testis B.

#### Experiment 2. Heritability of sperm length in *A. gambiae*

To determine whether the day of dissection influenced sperm length, a paired-t-test was used to compare the mean sperm length of the 64 males dissected day-2 post-emergence with that of their brothers dissected day-6 post-emergence. The variance components of mean sperm length and male wing length (see general statistical methods) were used to estimate the full-sib heritability of these two traits as follows:

h^2 ^= 2*σ^2^_family_/(σ^2^_family _+ σ^2^_plate _+ σ^2^_male_)

where σ^2^_family _is the variance in mean sperm length (or male wing length) among full-sib families, σ^2^_plate _is the variance among tissue culture plates within the full-sib families, and σ^2^_male _is the variance among males within the tissue culture plates.

A post-hoc power analysis was conducted to determine the power of experiment 2, which depends on the variance components of the five random factors (sperm, fields of view, males, tissue culture plates, families) and their sample sizes. For simplicity, only the effects of varying σ^2^_family _and the number of families were examined. To vary σ^2^_family_, the estimate from Table 1 (62 μm^2^) was multiplied by five ratios: 1.00, 1.25, 1.50, 1.75, and 2.00. The heritability (h^2^) of mean sperm length for these five values of σ^2^_family _were: 0.18, 0.27, 0.36, 0.45, and 0.54. These five h^2 ^values were cross-classified with nine family sample sizes: 32, 48, 64, 80, 96, 112, 128, 144, and 160. For each of the 45 combinations of h^2 ^and family sample size, 1000 replicates were run and the proportion of replicates with a significant σ^2^_family _was calculated (p < 0.05).

**Table 1:** Variance components of sperm and wing length in *A. gambiae *and *A. stephensi *males.

Experiment 1: *A. gambiae *sperm length (μm)							
Comp	df	SS	MS	V (μm^2^)	V (%)	F	P
male	24	3636431	151518	1012	10.7	4.98	< 0.001
testis	25	760304	30412	365	3.8	2.97	< 0.001
field	229	2345723	10243	206	2.2	1.30	0.002
sperm	2666	21031527	7889	7906	83.3		
total	2944	27773985		9489	100.0		
							
Experiment 1: *A. stephensi *sperm length (μm)							
Comp	df	SS	MS	V (μm^2^)	V (%)	F	P

male	16	1530523	95658	703	10.9	4.12	0.003
testis	17	394867	23227	275	4.3	3.05	< 0.001
field	178	1355025	7612	251	3.9	1.46	< 0.001
sperm	1854	9682493	5222	5221	80.9		
total	2065	12962908		6451	100.0		
							
Experiment 2: *A. gambiae *wing length (mm)							
Comp	df	SS	MS	V (μm^2^)	V (%)	F	P

family	31	0.58963	0.01902	3160.6	32.7	3.97	< 0.001
plate	32	0.153325	0.004791	0.0	0.0	0.65	0.911
male	64	0.45960	0.00741	6508.5	67.3		
total	127	1.202555		9669.1	100.0		
							
Experiment 2: *A. gambiae *sperm length (μm)							
Comp	df	SS	MS	V (μm^2^)	V (%)	F	p

family	31	1655126	53391	62	0.8	1.30	0.231
plate	32	1312648	41020	50	0.6	1.14	0.325
male	64	2308407	36069	577	7.4	4.99	< 0.001
field	512	3700502	7228	10	0.1	1.01	0.407
sperm	5760	41045231	7126	7126	91.1		
total	6399	50021914		7824	100.0		
							
Experiment 3: *A. gambiae *wing length (mm)							
Comp	df	SS	MS	V (μm^2^)	V (%)	F	p

family	15	0.071471	0.004765	140.8	3.1	1.06	0.416
male	44	0.197288	0.004484	4420.9	96.9		
total	59	0.268759		4561.8	100.0		
							
Experiment 3: *A. gambiae *sperm length (μm)							
Comp	df	SS	MS	V (μm^2^)	V (%)	F	p

family	15	338142	22543	0	0.0	0.84	0.634
male	45	1212059	26935	354	5.0	3.31	< 0.001
field	244	1985344	8137	150	2.1	1.23	0.012
sperm	2745	18216008	6636	6636	92.9		
total	3049	21751553		7140	100.0		

Experiments 1, 2, and 3: the variance in sperm length (μm) and male wing length (mm) was partitioned into various components (family, tissue culture plate, male, testis, field of view and sperm). For each component the degrees of freedom (df), sum of squares (SS), mean square (MS), the variance (V) with units of μm^2 ^or as a percent (%), the F-statistic (F), and the statistical significance (p) are shown.

The phenotypic and genetic correlations between sperm length and wing length were estimated. The genetic correlation between sperm length and wing length was estimated as the correlation between family means [27].

#### Experiment 3. Sperm length and male reproductive success in *A. gambiae*

For each of the 16 sire families, three sire fitness traits were measured on the females mated to that sire family: the proportion of inseminated females (insemination success), the proportion of females with at least one motile sperm in their spermathecae (sperm motility), and the proportion of ovipositing females (oviposition success). In addition, for each of the 16 sire families, two sire morphological traits were measured (on four haphazardly selected males): the mean sperm length and the mean wing length. The correlation matrix for these five sire traits was calculated.

Insemination success, sperm motility and oviposition success are binomial data. The generalized linear model (GLM) function in R, glm, with a binomial error term was used to model each of the three fitness traits as a function of mean sperm length, mean wing length, and their interaction for the 16 sire families. To check the fit of the GLM models to the data, the residual degrees of freedom were compared to the residual deviance. Log likelihood-ratio tests and the Chi-square distribution were used to determine the statistical significance of the terms in the GLM. To interpret the parameter estimates, sperm length and wing length were transformed to z-scores (mean = 0, standard deviation = 1). This transformation allows comparison of the slopes (B_1 _and B_2_) from the GLM, fitness ~B_0 _+ B_1_(z.sperm) + B_2_(z.wing), because the z-transformed sperm (z.sperm) and wing lengths (z.wing) are measured in the same units (standard deviations). The slopes from these GLMs measure the strength of selection on sperm length and male wing length [28]. To compare the effects of sperm length and wing length, the percent change in fitness was calculated when one trait was increased by one standard deviation while the other was held constant.

## Results

### Experiment 1. Intra-specific variation in sperm length in *A. gambiae *and *A. stephensi*

The mean sperm length was significantly correlated between the two testes in both *A. gambiae *(t = 4.32, df = 23, r = 0.67, p < 0.001) and *A. stephensi *(t = 3.23, df = 15, r = 0.64, p = 0.006; Figure 1). The mean sperm length of some *A. gambiae *males (250 μm) was 2.5 times longer than that of others (100 μm; Figure 1). In both *A. gambiae *and *A. stephensi*, differences among males accounted for ~11% of the total variance in sperm length (Table 1). Hence there was intra-specific variation in sperm length in both *A. gambiae *and *A. stephensi*. The mean sperm length (± standard error) of *A. gambiae *was 197 ± 7.2 μm and that of *A. stephensi *was 235 ± 7.4 μm.

### Experiment 2. Heritability of sperm length in *A. gambiae*

The mean sperm length (± standard error) of the 128 males was 202 ± 2.9 μm. The mean sperm length of the 64 males dissected on day-2 post-emergence (201 ± 3.4 μm) was not significantly different from that of their brothers dissected on day-6 post-emergence (204 ± 3.8 μm; t = -0.49, df = 63, p = 0.629). The variance components due to differences among families, tissue culture plates, males, fields of view, and individual sperm accounted for 0.8%, 0.6%, 7.4%, 0.1%, and 91.1%, respectively, of the total variance in sperm length (Table 1). The variance component due to differences among males was statistically significant (p < 0.001; Table 1). After calculating the mean sperm length for each of the 128 males, the full-sib heritability of mean sperm length was 0.18.

**Figure 2:**
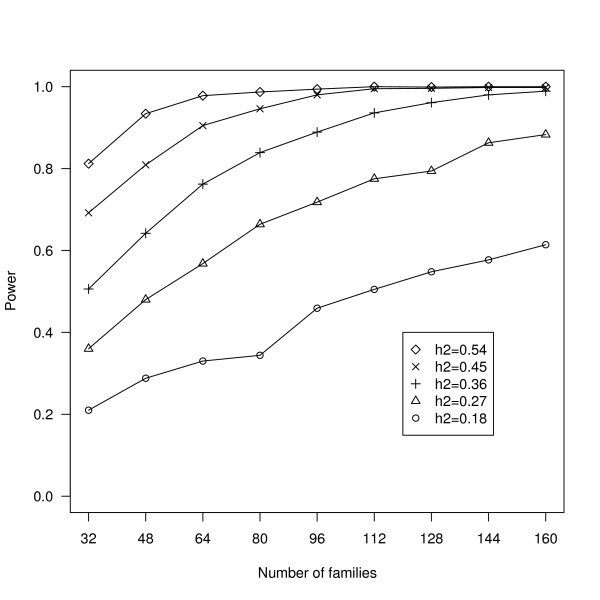
**The retrospective power analysis of the heritability of mean sperm length in *A. gambiae***. Experiment 2: for each of five heritabilities of mean sperm length in *A. gambiae *(0.18, 0.27, 0.36, 0.45, 0.54) and the nine family sample sizes (32, 48, 64, 80, 96, 112, 128, 144, 160), the power to detect a statistically significant among family variance component in mean sperm length is shown.

For the observed heritability of mean sperm length in *A. gambiae *(h^2 ^= 0.18), the post-hoc power analysis found that the power of experiment 2 to detect a statistically significant among family variance component in mean sperm length (σ^2 ^_family_) was 20% (Figure 2). After quintupling the family sample size (n = 160 families), the power of experiment 2 to detect a significant σ^2 ^_family _was 60% (Figure 2). When the heritability of mean sperm length was tripled from 0.18 to 0.54, the power of experiment 2 to detect a significant σ^2 ^_family _was 80% (Figure 2).

The mean wing length (± standard error) of the 126 *A. gambiae *males was 3.07 ± 0.012 mm. The variance components due to differences among families, tissue culture plates, and males accounted for 32.7%, 0.0%, and 67.3%, respectively, of the total variance in male wing length (Table 1). The variance component due to differences among families was statistically significant (p < 0.001; Table 1), but that due to differences among plates was not (p = 0.911; Table 1). The full-sib heritability of male wing length was 0.65.

**Figure 3:**
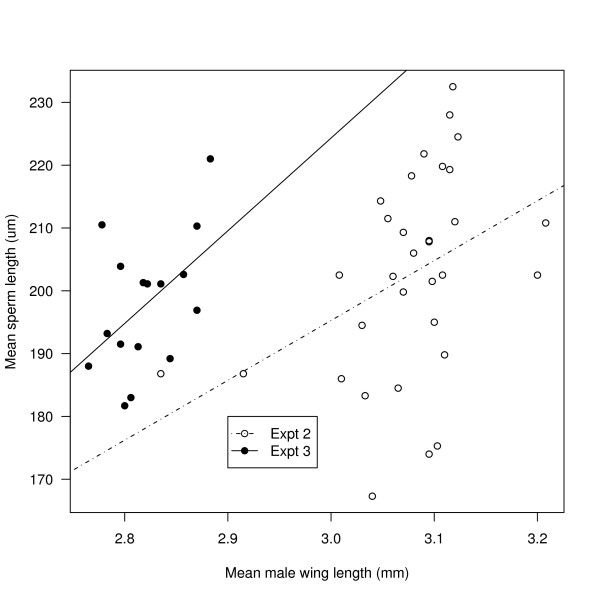
**The correlation between sperm and wing length in *A. gambiae *males**. Experiments 2 and 3: sperm length increases with male wing length in *A. gambiae*. Shown are the means for the 32 families in experiment 2 (open circles) and the 16 families in experiment 3 (filled circles). The lines of best fit for experiments 2 and 3 are shown with the stippled and solid lines, respectively.

Across the 126 males, sperm length increased with wing length and this phenotypic correlation was almost statistically significant (r = 0.16, t = 1.76, df = 124, p = 0.081). Across the 32 families, sperm length increased with wing length and this genetic correlation was statistically significant (r = 0.40, t = 2.41, df = 30, p = 0.022; Figure 3).

In summary for experiment 2, there was intra-specific variation in sperm length in *A. gambiae*. There was no evidence for genetic variation in sperm length in the Keele population of *A. gambiae *although the power was low (20%). The heritability of sperm length was low (h^2 ^= 0.18) compared to the heritability of wing length (h^2 ^= 0.65). The phenotypic (r = 0.16) and genetic (r = 0.40) correlations between sperm length and wing length were both positive and the latter was statistically significant.

### Experiment 3. Sperm length and male reproductive success in *A. gambiae*

There were three males from the same family that did not have any discernible testes. Hence the mean sperm length was measured on 64 – 3 = 61 males. The mean sperm length (± standard error) of these 61 males was 198 ± 2.9 μm. The variance components due to differences among families, males, fields of view, and individual sperm accounted for 0.0%, 5.0%, 2.1%, and 92.9%, respectively, of the total variance in sperm length (Table 1). The variance component due to differences among individual males was statistically significant (p < 0.001; Table 1). The full-sib heritability of mean sperm length was less than 0.01.

The mean wing length (± standard error) of the 60 males was 2.82 ± 0.009 mm, which was 8.1% smaller than that in experiment 2. This difference in wing length between experiments 2 and 3 was most likely caused by differences in larval rearing. The larvae in experiment 2 were reared individually in tissue culture plates as soon as they hatched whereas the larvae in experiment 3 spent two days at high density in their communal family containers before being transferred to tissue culture plates. The variance components due to differences among families, and males accounted for 3.1%, and 96.9%, respectively, of the total variance in male wing length (Table 1). The variance component due to differences among families was not statistically significant (p = 0.416; Table 1). The full-sib heritability of male wing length was 0.06.

Across the 60 males, sperm length increased with wing length and this phenotypic correlation was statistically significant (r = 0.38, t = 3.16, df = 58, p = 0.003). Across the 16 families, sperm length increased with wing length and this genetic correlation was almost statistically significant (r = 0.49, t = 2.12, df = 14, p = 0.052; Figure 3).

**Figure 4:**
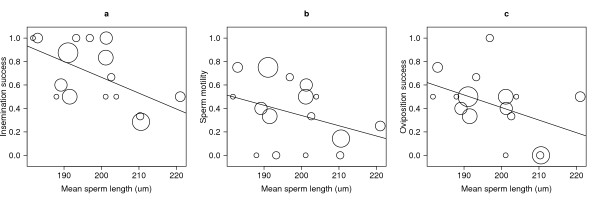
**The correlation between sperm length and reproductive success in *A. gambiae *males**. Experiment 3: The proportion of inseminated females (insemination success; a), the proportion of females with at least one motile sperm in their spermathecae (sperm motility; b) and the proportion of ovipositing females (oviposition success; c) decrease with the sire family's mean sperm length in *A. gambiae*. The sizes of the circles represent the number of females in the denominator of the proportion. The lines of best fit are shown for the 16 sire families.

Of the 65 females that survived to the end of the oviposition period, 69.0% were inseminated (45/65), 40.0% had at least one motile sperm in their spermatheca (26/65), and 41.5% oviposited (27/65). Across the 16 mating cages, the three sire fitness traits: insemination success, sperm motility, and oviposition success were all positively and significantly correlated with each other (Table 2). The three male fitness traits were all negatively correlated with the sire family's mean sperm length (Figure 4) but the correlation was only statistically significant for the proportion of inseminated females (Table 2). The three male fitness traits were not correlated with the sire family's mean wing length (Table 2).

**Table 2:** The correlation matrix of five traits in *A. gambiae *males.

Three male fitness traits and two male morphological traits					
Trait	p.insem	p.motile	p.ovip	wing	sperm
p.insem	***	**0.63**	**0.74**	-0.05	**-0.55**
p.motile	**0.009**	***	**0.58**	0.09	-0.34
p.ovip	**0.001**	**0.017**	***	0.00	-0.43
wing	0.843	0.739	0.991	***	0.49
sperm	**0.026**	0.191	0.101	0.050	***

Experiment 3: the correlation matrix for the three sire fitness traits: the proportion of inseminated females (p.insem), the proportion of females with at least one motile sperm in their spermathecae (p.motile), and the proportion of ovipositing females (p.ovip), and the two sire morphological traits: mean wing length (wing) and mean sperm length (sperm) is shown for the 16 sire families. The Pearson's correlation coefficient and its p-value are shown above and below the diagonal, respectively. Statistically significant correlations and p-values are shown in bold.

For the GLM analysis, the residual degrees of freedom were similar to the residual deviance indicating that the GLM models were a good fit to the data (Table 3). The GLM analysis found that all three male fitness traits decreased significantly with the mean sperm length of the sire family but that there was no significant effect of the sire family's mean wing length (Table 3). The partial logistic regression coefficients show that increasing either sperm length or wing length by one standard deviation changed oviposition success by -39% and +31%, respectively (Table 4).

**Table 3:** Reproductive success as a function of sperm and wing length in *A. gambiae *males (GLM models)

model id	model	res df	res dev	AIC
1	p.insem ~ sperm * wing	12	16.59	44.38
2	p.insem ~ sperm + wing	13	16.60	42.40
3	p.insem ~ wing	14	23.84	47.63
**4**	**p.insem ~ sperm**	**14**	**17.97**	**41.77**
5	p.insem ~ 1	15	23.88	45.68
				

effect	comparison	Δdf	Δdev	p
sperm*wing	1 vs. 2	1	0.02	0.903
**sperm**	**2 vs. 3**	**1**	**7.23**	**0.007**
wing	2 vs. 4	1	1.37	0.242
				

model id	model	res df	res dev	AIC
6	p.motile ~ sperm * wing	12	15.38	45.83
7	p.motile ~ sperm + wing	13	15.96	44.41
8	p.motile ~ wing	14	21.02	47.47
**9**	**p.motile ~ sperm**	**14**	**17.34**	**43.79**
10	p.motile ~ 1	15	21.16	45.61
				

effect	comparison	Δdf	Δdev	p
sperm*wing	6 vs. 7	1	0.58	0.447
**sperm**	**7 vs. 8**	**1**	**5.06**	**0.025**
wing	7 vs. 9	1	1.38	0.240
				

model id	model	res df	res dev	AIC
11	p.ovip ~ sperm * wing	12	14.42	44.90
**12**	**p.ovip ~ sperm + wing**	**13**	**15.56**	**44.05**
13	p.ovip ~ wing	14	21.24	47.72
14	p.ovip ~ sperm	14	17.99	44.47
15	p.ovip ~ 1	15	21.73	46.21
				

effect	comparison	Δdf	Δdev	p
sperm*wing	11 vs. 12	1	1.15	0.284
**sperm**	**12 vs. 13**	**1**	**5.68**	**0.017**
wing	12 vs. 14	1	2.43	0.119

Experiment 3: GLM with binomial errors was used to model the proportion of inseminated females (p.insem), the proportion of females with at least one motile sperm in their spermathecae (p.motile), and the proportion of ovipositing females (p.ovip) as a function of the sire family's mean sperm length (sperm), the mean wing length (wing), and their interaction (sperm*wing). Shown for each model are the residual degrees of freedom (res df), the residual deviance (res dev), and the Akaike information criterion (AIC). Shown for the log likelihood ratio tests of the effects are the change in the degrees of freedom (Δdf), the change in the residual deviance (Δdev), and the statistical significance of the effect (p). The best model according to AIC and the statistically significant effect sizes are shown in bold.

**Table 4:** Reproductive success as a function of sperm and wing length in *A. gambiae *males (Parameter estimates)


model 2: p.insem ~B_0 _+ B_1_(z.sperm) + B_2_(z.wing)				
param	estimate	s.e.	Mean fitness (p.insem)	% Δ
B_0_	0.95	0.297	exp(B_0_)/(1+exp(B_0_)) = 0.72	
B_1_	-0.84	0.330	exp(B_0 _+ B_1_)/(1+exp(B_0 _+ B_1_)) = 0.53	-27.0
B_2_	0.38	0.328	exp(B_0 _+ B_2_)/(1+exp(B_0 _+ B_2_)) = 0.79	9.6
				

model 7: p.motile ~B_0 _+ B_1_(z.sperm) + B_2_(z.wing)				
param	estimate	s.e.	Mean fitness (p.motile)	% Δ
B_0_	-0.41	0.265	exp(B_0_)/(1+exp(B_0_)) = 0.40	
B_1_	-0.69	0.325	exp(B_0 _+ B_1_)/(1+exp(B_0 _+ B_1_)) = 0.25	-37.3
B_2_	0.39	0.337	exp(B_0 _+ B_2_)/(1+exp(B_0 _+ B_2_)) = 0.49	24.0
				

model 12: p.ovip ~B_0 _+ B_1_(z.sperm) + B_2_(z.wing)				
param	estimate	s.e.	Mean fitness (p.ovip)	% Δ
B_0_	-0.34	0.265	exp(B_0_)/(1+exp(B_0_)) = 0.42	
B_1_	-0.73	0.329	exp(B_0 _+ B_1_)/(1+exp(B_0 _+ B_1_)) = 0.25	-38.8
B_2_	0.52	0.343	exp(B_0 _+ B_2_)/(1+exp(B_0 _+ B_2_)) = 0.54	30.9

The parameters (B_0_, B_1_, and B_2_) and their estimates and standard errors (s.e.) from GLM models 2, 7 and 12 in Table 3 after standardizing the sire family's mean sperm length (z.sperm) and mean wing length (z.wing) to z-scores. The logit-link function was used to back-calculate the mean fitness (p.insem, p.motile, p.ovip) when the average sire family (z.sperm = 0, z.wing = 0) increased its mean sperm length by one standard deviation while mean wing length was held constant (z.sperm = 1, z.wing = 0), and vice versa (z.sperm = 0, z.wing = 1). The percent change in fitness (% Δ) was calculated relative to the mean fitness of the average sire family.

In summary for experiment 3, there was intra-specific variation in sperm length in *A. gambiae*. The heritabilities of sperm length (h^2 ^< 0.01) and wing length (h^2 ^= 0.06) were low. The phenotypic (r = 0.38) and genetic (r = 0.49) correlations between sperm length and wing length were both positive and the former was statistically significant. The three measures of male reproductive success all decreased significantly with sperm length but there was no effect of male wing length. Males with shorter sperm have greater reproductive success.

## Discussion

This study found intra-specific variation of sperm length in both *A. gambiae *and *A. stephensi *(Figure 1). Differences among *A. gambiae *males accounted for a significant portion of the variance in sperm length in all three experiments (Table 1). In experiment 2, there was no genetic variation in sperm length in the Keele population of *A. gambiae *(Table 1), but sperm length was genetically correlated with male wing length (Figure 3). According to the GLM (Table 3, Figure 4), the three sire fitness traits: insemination success, sperm motility, and oviposition success all decreased significantly with the sire family's mean sperm length, but there was no effect of the sire family's mean wing length. One critique of experiment 3 is that the sire family is confounded with the dam family. Hence, variation in the three measures of reproductive success may be caused by differences among sire families, differences among dam families, or interactions between sire and dam families. Regardless, it is still true that for each unique combination of sire and dam family, reproductive success decreased significantly with the sperm length of the sire family. The mean sperm length of *A. gambiae *was very similar among experiments 1, 2, and 3 (197, 202, and 198 μm, respectively) and was considerably shorter than the estimate from the Klowden and Chambers [17] study (~280 μm). The two studies are difficult to compare because Klowden and Chambers [17] measured ~100 sperm on three to five males whereas in the present study 12100 sperm on 217 males were measured.

This is the first study to report intra-specific variation in sperm length among males in a species of mosquito. Klowden and Chambers [17] showed that *A. gambiae *males had more variable sperm than other anopheline species, but their study did not estimate variation among males. For *A. gambiae *in the present study, the mean sperm length among males ranged between 100 and 250 μm (Figure 1). However, most of the variance in sperm length occurred within males (i.e. 89%, 91%, and 97% in experiments 1, 2, and 3). This means that each male has sperm with a great variety of lengths. By contrast, in *Drosophila mojavensis*, the variance in sperm length among males is over three times greater than that within males [29]. The power to detect genetic variation in mean sperm length among families was low (20%). The estimate of the full sib heritability of mean sperm length in this study was much lower (h^2 ^= 0.18) than what has been reported in the literature [19,20]. If the heritability of mean sperm length in *A. gambiae *was similar to *G. bimaculatus *[h^2 ^= 0.52; 20] or *S. stercoraria *[h^2 ^= 0.67; 19], this study would have had sufficient power (Figure 2). One obvious explanation for the low heritability of sperm length in *A. gambiae *is the large variance within males. Another explanation is polyandry (i.e. families had a mixture of full-sibs and half-sibs) which, although rare in the field [10], is more common in laboratory populations [~24%; 30]. For future work, the power analysis suggests quintupling the number of families (~240 hours of work; Figure 2). The variance component analysis (Table 1) suggests shifting the sampling effort from factors that did not influence variation in sperm length (tissue culture plates and fields of view) to those that did (males and individual sperm). Sperm clumping was a problem in this study (see methods) and sampling bias might have occurred, if sperm of a certain length were more likely to clump. Future work on sperm length in *A. gambiae *should search for a chemical or technique that can reduce clumping. Other possible explanations for the low heritability of mean sperm length include founding effects and laboratory selection. During the colonization process, there is an inevitable loss of genetic variation in *Anopheles *colonies as many individuals do not mate under laboratory conditions [12]. The Keele strain used in this study, despite being outbred [see [26]], might have less genetic variation for sperm length than wild populations, even if the colonization process maintained the genetic variance for wing length. Future quantitative genetic experiments on sperm length should therefore focus on wild populations of *A. gambiae*.

The importance of the environment on the heritability of quantitative traits is shown by the 10-fold difference in the full-sib heritability of wing length between experiments 2 and 3 (0.65 versus 0.06). Differences in sample size between experiments 2 and 3 (32 versus 16 full-sib families) cannot account for this discrepancy because the variances on which the heritabilities are based are independent of sample size. One explanation is that in experiment 3, the larvae spent two days at high density (see materials and methods) and heritabilities are often lower in sub-optimal environments [31,32]. The full-sib heritability of wing length in experiment 2 (h^2 ^= 0.65) was higher than that in a field-captured population of *A. gambiae *(h^2 ^= 0.35; [33]), illustrating that quantitative genetic variation in a laboratory colony is not always lower than that in a wild population.

The discovery that sperm length was negatively correlated with male reproductive success in *A. gambiae *suggests that sperm length is an important measure of male fitness and represents a novel contribution to understanding the reproductive biology of this medically important vector. The results in this study are consistent with two other species of insect: *G. bimaculatus *[24] and *O. taurus *[25], where males with shorter sperm have higher fertilization success. Why do males with shorter sperm have higher reproductive success in *A. gambiae*? Life-history theory suggest that there are trade-offs between sperm quantity (number of sperm) and sperm quality (length, viability, swimming speed), although only a few studies have found evidence for such trade-offs [21]. For example, of the 11 studies investigating a negative correlation between sperm number and sperm length [21], only three found the expected trade-off in *Drosphila *fruit flies [34], the snail, *Vivaparus ater *[35], and the yellow-pine chipmunk, *Tamias amoenus *[36]. Klowden [30] recently showed in *A. gambiae *that innervations from a sperm-filled spermatheca cause females to switch to the mated state (rather than male accessory gland fluids [37,38] which are well known to inhibit female multiple mating in the yellow-fever mosquito, *Aedes aegypti *[39,40]). This suggests that the volume of sperm transferred to fill the female spermatheca is important for inducing female oviposition behavior [30]. Hence, males producing lots of short sperm may be better at filling a female's spermatheca and inducing oviposition than males producing a few large sperm.

The finding that males with short sperm have higher reproductive success appears to contradict the work of Klowden and Chambers [17]. They found that the mean sperm length in the female reproductive tract and spermatheca was significantly longer than that in the male testes. This suggests that males with longer sperm have higher reproductive success. However, these two results are not necessarily mutually exclusive. In *A. gambiae*, each male has a great variety of sperm lengths. Males that produce lots of short sperm also produce long sperm. As observed in other insects [41,42], it may be that short sperm act as filler that signal to the female that she is mated whereas long sperm fertilize the eggs. For example, *Drosophila pseudoobscura *males produce both long (300 μm) and short (75 μm) sperm but only the long sperm fertilize the eggs [42]. Similarly in the Lepidoptera (butterflies and moths), males produce long nucleated (eupyrene) sperm that fertilize the eggs and short anucleated (apyrene) sperm that are believed to act as filler to prevent female re-mating [41]. In contrast to the two distinct sperm lengths in *D. pseudoobscura *[42] and the apyrene sperm in the Lepidoptera [41], *A. gambiae *males produce a continuous distribution of sperm lengths (this study) and Klowden and Chambers [17] showed that all of these sperm are nucleated and should therefore be able to fertilize the egg. Finally, it is difficult to compare the present study to that of Klowden and Chambers [17] due to differences in methodology. They used different males to estimate the distribution of sperm lengths in the female reproductive organs and the male testes. They did not show that females with longer sperm had higher oviposition success. Their study suggests selection on sperm length within a male's ejaculate whereas this study measured selection on sperm length among groups (full-sib families) of males.

Selection experiments with *Drosophila melanogaster *have shown that male fertilization success is determined by an interaction between sperm length and the length of the female sperm storage organ [43]. Males selected for long sperm had higher fertilization success than males selected for short sperm when mating with females selected for long sperm storage organs, but there was no difference in fertilization success between long-sperm and short-sperm males when mating with females selected for short-sperm storage organs [43]. Conversely in *O. taurus*, males with short sperm had the highest fertilization success when mating with females with medium- to large-sized spermathecae whereas males with intermediate-sized sperm had the highest fertilization success when mating with females with small spermathecae [25]. Across five different anopheline species, sperm length appears to be positively correlated with spermatheca volume [17] suggesting that female morphology is exerting selection on sperm length as shown in other taxa [21].

Selection on sperm length was not caused by correlated selection on body size. Sperm length and male wing length were positively correlated (Figure 3), but selection on these two traits was in opposite directions and was not statistically significant for wing length (Table 4). In this study, male body size was not important for male reproductive success, perhaps because all the males were reared under the same conditions, so that there was little variation in male body size. In contrast, other laboratory studies have found that *A. gambiae *males that captured a female during swarming were slightly larger (mean wing lengths = 2.82 versus 2.76 mm) than those that did not [44], although a more recent study found that intermediate-sized males were the most successful at capturing females during swarming [45]. In a wild population of *A. freeborni*, large males mated more often than smaller ones as revealed by examining their accessory glands [8]. Male body size and sperm length most likely influence different components of male reproductive success. Large males may be more successful at acquiring mates, whereas males with many short sperm are more successful at inducing females to oviposit.

The present study does not support the conclusion that sperm length is causal with respect to oviposition success in *A. gambiae*. Males with short sperm may produce more sperm or more accessory gland fluids than males with long sperm. Female factors such as the size of her reproductive tract or spermatheca, the nutritional value of the male accessory gland fluids and the blood meal (i.e. the level of resources available for egg production), or her tendency to oviposit in the laboratory environment could all change the relationship between sperm length and oviposition success. Furthermore, in experiment 3, sperm length was measured in the males four days after they were separated from the females. Hence an alternative explanation for the negative relationship between male sperm length and oviposition success is that mated males transferred their long sperm to the females and had not replenished their long sperm supplies by the time they were assayed for sperm length. Previous work on a multiply mated laboratory population of *A. culicifacies *found that male reproductive success peaked at day-3 and day-7 post-emergence separated by four days of rest [46]. In *A. stephensi*, males that were given continuous access to virgin females had "rest periods" of up to three days in between fertilizing females [12]. Similarly, Voordouw *et al *[16], using lines of *A. gambiae *derived from this study's Keele population, found that male reproductive success was correlated between two days of mating separated by one day of rest. These three studies suggest that the males in the present study were given sufficient time (four days) to replenish their sperm supplies. Unfortunately, it is not possible to measure male sperm length before mating, because the procedure kills the male. However, it is possible to measure sperm length in the spermatheca of the female and future experiments should investigate whether *A. gambiae *females that oviposit are more likely to have long sperm in their spermathecae than females that do not.

## Conclusion

This is the first study to demonstrate that there is significant intra-specific variation in sperm length in *A. gambiae*. *Anopheles gambiae *males with short sperm had significantly higher reproductive success than males with longer sperm and this is important for any strategy considering the release of transgenic males.

## Competing interests

The authors declare that they have no competing interests.

## Authors' contributions

MJV conceived the idea, designed and ran the experiment and analysed the data. MJV, JCK and HH interpreted the data and wrote the paper.
